# In the Air of the Natural History Museum: On Corporate Entanglement and Responsibility in Uncontained Times

**DOI:** 10.1007/s10978-020-09280-w

**Published:** 2020-10-21

**Authors:** Lilian Moncrieff

**Affiliations:** School of Law, University of Glasgow, Glasgow, G12 8QQ UK

**Keywords:** Anthropocene, Corporate responsibility, Materialism, Natural history

## Abstract

This paper discusses corporate entanglement, impactfulness and responsibility in the Anthropocene, amidst events and conditions that ‘uncontain’ time. It takes its direction of travel from artist Brian Jungen’s ‘Cetology’ (2002), a whalebone sculpture made out of cut-up plastic garden chairs, which conjoins the times of earth and world history, as it hangs in the air of the art gallery, ‘as if’ exhibited in the natural history museum. The paper relates ‘Cetology’s’ engagement with natural history, time, and commodification to matters of corporate entanglement and responsibility within company law and governance. Problems with understanding the comparable imprint of large and multinational companies on matters, places, and communities are identified, after the domination of corporate legal frameworks over nature and the stability and perfection of economic incentives at law. The reading of this (critical-legal) situation is developed through theory and engagement with materialist and critical thinkers, who unite in their concern with distributed human–nature relations and the ‘concreteness’ that attends collisions in time, ruin and affect. Walter Benjamin and Theodor Adorno’s writings are central to this analysis, and strengthen the commentary on ‘natural history’ themes curated by Jungen. The paper contemplates how times’ uncontainment might invoke a change in expectation and method for the company law field, assigning contingency to the corporation and provoking a new mode of reflection about corporate entanglement and responsibility at law.

## Introduction

This paper discusses corporate entanglement, impactfulness and responsibility in uncontained times. The latter term (uncontained times) expresses the interest of the author in thinking corporate law and governance for the world impacted, over generations, by industry, commerce and globalisation and for the epoch of the Anthropocene.^1^ This is where material migrations of human affect and activity, and their cumulative impact on nature and people, have become as pertinent (normatively, politically, operationally, economically) to businesses and the future of corporate institutions as the linking up of regions, organisations, technologies and markets.^2^ Mounting anthropogenic entanglements create a situation where there is ‘a constant conceptual traffic between Earth history and world history’, and the interconnection of ‘events that happen on vast geological scales—such as changes to the whole climate system of the planet—with what we might do in the everyday lives of individuals, collectivities, institutions, and nations’ (Chakrabarty 2018, p. 6). Historian Dipesh Chakrabarty highlights how these earthly entanglements ‘outscale our very human sense of time’, and inaugurate a perspectival shift in the consciousness of change and responsibility or duty: ‘It is as if the Earth system were saying to the conscious part of its constituents, humans … “you never look at me from the place from which I see you”’ (ibid. p. 29).

The paper’s focus on corporate entanglement and responsibility fits with this special issue’s effort to reassess legal ‘theories and frameworks that have prevailed within dominant accounts of law, rights, governance, sovereignty and the ordering of associative life’ (Birrell and Matthews 2020). It proceeds by situating companies and commerce within ‘the entangled sphere of the relations that the Anthropocene has illuminated’ (ibid.) and in uncontained times. The paper observes how efforts to cultivate responsibility within corporate legal orders currently extend the self-governance of companies and market order, as a means of ‘managing’ diffuse entanglements.^3^ Yet by doing so current policies would also seem to pull back from the ‘disturbed certainties’ (Chakrabarty 2018, p. 31), which attend the integration of geological and human time-scales, and to affirm the place of *companies* at the centre of world history instead. The paper, concerned about this approach, explores the possibility that the histories coming to light might better ‘de-center’ the corporation and dramatise its interconnectedness, as a legal personality that intersects with a huge range of existents (human, non-human) and experiences that bear upon justice and equality. It explores the development of companies’ actions and impactfulness among ‘matters’,^4^ places and communities, and the confinement of some impacted existents to the margins. It thinks, further, about routes out of this (confinement) and about how temporal mutinies might bear upon and renew expectations for the corporate legal field, from and for the margins.

In terms of method, the paper begins by encountering Brian Jungen’s sculptural work, ‘Cetology’ (2002) in the art gallery. The analysis serves to highlight the implication of commodity trails, relatable to economic actors, and to comment on industrial histories that juxtapose the different times of earth and world history. Critical methods are used in Part II to relate this dis-containment of time to the field of company law, and to describe and assess the current drive to corporate responsibility and sustainability in UK law and governance. Problems with understanding earth history and the imprint of multinational companies on matters, places, and communities over time are identified, after the domination of corporate legal frameworks over nature and the stability and perfection of economic incentives at law. The reading of this (critical-legal) situation is developed, in Part III, through theory and engagement with materialist and critical thinkers, united in their concern with distributed human-nature relations and with the concrete impacts that attend collisions in time, ruin and affect. Walter Benjamin and Theodor Adorno’s writings are central to this analysis, and strengthen the commentary on ‘natural history’ themes, curated by Jungen. Part IV offers, finally, to clarify the paper’s interest in substituting the naturalness of the company, and also management, with spontaneous concern for the affected and for existences that lie within horizons of ruin and disorder at law. The paper contemplates how times’ uncontainment could and should invoke a change in expectation and method for the company law field, assigning more contingency to the corporation and provoking a new mode of reflection about corporate entanglement and responsibility at law.

## I

**Fig. 1:**
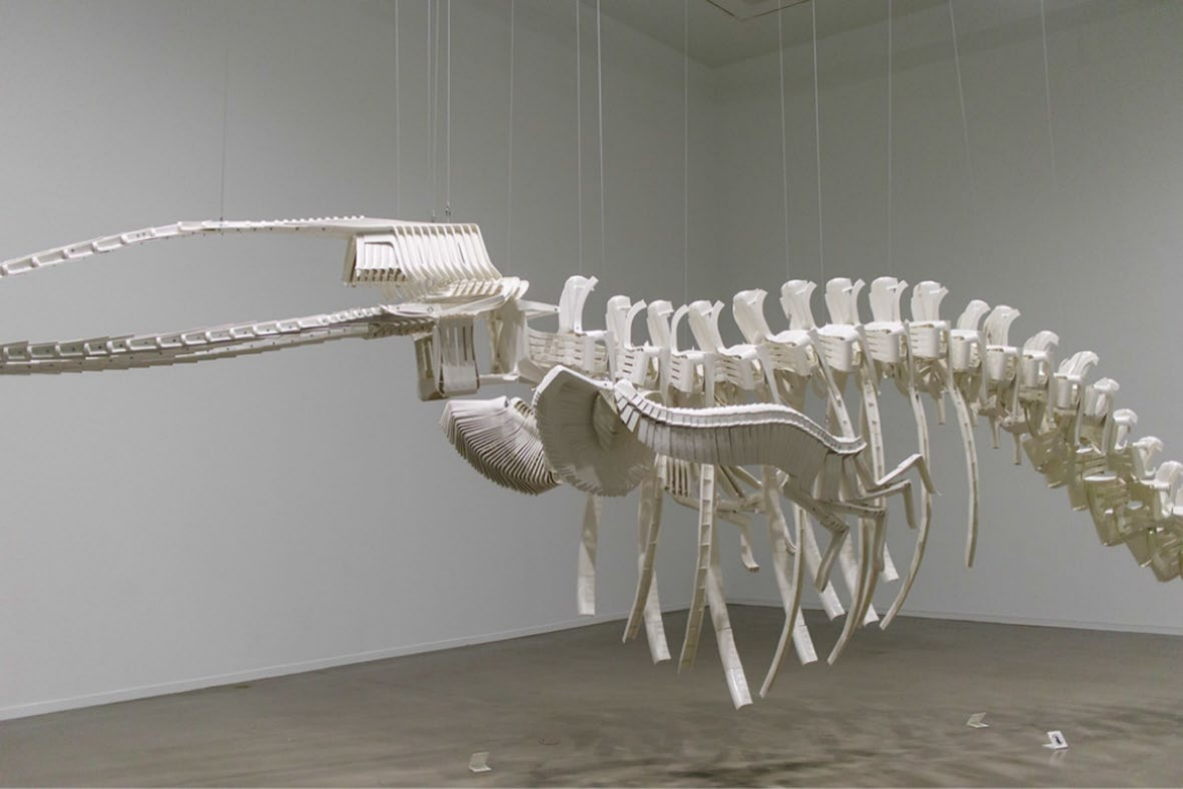
*Cetology*—Brian Jungen. Image by ¡Carlitos (Creative Commons license, available to view at https://search.creativecommons.org/photos/9760d400-8149-4f26-aae2-2593b6926284 (accessed 17 September 2020))

This is ‘Cetology’ (Fig. 1), a monumental whalebone sculpture conceptualised, fabricated and exhibited in the Vancouver Art Gallery by Canadian and Dane-zaa artist, Brian Jungen (2002). The sculpture is suspended in the air of the art gallery, as if hanging in the classic style of a natural history museum exhibit. Closer inspection reveals, however, that the work is not fashioned out of natural materials or fossilised mammal bones. It is, in fact, cut out of plastic (polypropylene) patio chairs, a mass manufactured product, redeployed and made unfamiliar in Jungen’s work. (The artist has a reputation for experimenting with commercial products to create unfamiliar constructions; he has previously worked with golf bags, Nike running shoes, freezers and sofas.)

Jungen’s work captures the cultural significance and majesty of nature alongside a strong sense of the environmental and existential dangers that face marine life and some (human, non-human) communities in the context of climate change, oceans of plastic, pollution, commercial hunting and overfishing. The sculpture registers a dramatic change in what societies might monumentalize in the Anthropocene, as man-made materials, like polypropylene, take on existences and entanglements *separate* to their product and commercial existence.^5^ This is where artefacts, like the patio chairs that Jungen cuts up, ‘live on’ and exist beyond their ‘usefulness’ to companies and consumers (Brown 2015, p. 206). This ‘living on’ happens where the decomposition of plastics and industrial materials, used in the chairs, reaches upwards of hundreds of years, and generates entanglements that reach into human and non-human food chains, soil structures, ocean beds, and (ultimately) mineralized fossil records. It acquires historical dimensions, following Christopher Tomlin’s conceptualisation of ‘law’s objects’ and *Fortleben,*^6^ as ‘the soul of the original that is past (its once-was), its afterlife that survives in translation, and its living on that moves elsewhere’ (Tomlins 2018, p. 7). In a reference to this separate and also double existence (commercial, material), some of the chairs that Jungen used for the sculpture were put into production with the Canadian Tire price tags still on.^7^ ‘Cetology’, as such, and reaching for accolades that Theodor Adorno once conferred on Walter Benjamin, awakens ‘congealed life in petrified objects—as in allegory’. He encourages the gallery visitor (turned natural history museum visitor) to ‘scrutinize living things so that they present themselves as being ancient, “ur-historical” and abruptly release their significance’ (Adorno 1983, p. 233).^8^

Jungen’s work is interesting, for this paper and issue, for where it leads its visitors quizzically and contemplatively through an unexpected experience of plastic, natural-cultural entanglement, and through the intended and unintended consequences of commerce and modernity. Theodor Adorno in his 1932 (but posthumously published) lecture concerning the ‘idea of natural history’ (1984) also fascinated over this consequential horizon, where the diffuse elements of nature and history ‘break apart and interweave at the same time in such a fashion that the natural appears as a sign for history and history’ (ibid. p. 121). The lecture identified ‘natural history’ as a problem that ‘from the perspective of philosophy presents itself first as the question of how it is possible to know and interpret an alienated, reified, dead world’ (ibid. p. 118), of ‘things created by man, yet lost to him’ (ibid. p. 117). Adorno’s wider writings (with Horkheimer 1997) would eventually explain the philosopher’s views on the fate of lost and adventitious elements in commercial societies, and how they became ‘disenchanted’ or ‘alienated’ after the modern drive to human mastery over nature (in science, reason, economy, etc.). It is, however, in this earlier lecture that Adorno expressed his interest in creating political-normative *encounters* with existences thrown into such a state of ruin, and onto the natural history register. He speaks about his determination to bring mortified and estranged natures ‘out of infinite distance’ and ‘into infinite closeness’, as objects of philosophical interpretation (ibid. p. 119). He cites (admiringly, again) the work of his peer and fellow philosopher, Benjamin, as the turning point for realising this ambition, and for reading history on the surfaces of the surviving, as the ‘register of the trace’. We learn about progress, in this reading, not by trying to fit the alienated and discounted (or their ‘living on’) into ‘general structures’, but in ‘paradigms’ and ‘as interpretations of concrete history’, which reveal themselves on the surfaces of objects, in ruin and in transience (ibid. p. 119). Ruin invokes the ‘structure of inner-historical events’ (ibid. p. 117),^9^ and suggests reading fragmented matters in ‘an upward direction at every single one of its points’, for which, ‘allegory is the key mode of expression’ (ibid. p. 119).

‘Cetology’, also, philosophically dis-contains temporal compressions and castigations. Price tags sit furtively within the bones of the whale structure, accounting for the stealth of industry, clocks, for semiautomatic trading platforms and all the rest. But none—tags, clocks, calculators—take control of history. Jungen, instead, *juxtaposes* commodity histories (price tags on) and the incalculable histories that lie before and after production (the manufacturing demand on ecosystems, cultural homogenisation, waste and ruin). As allegorist, Jungen takes a break from ‘the affirmative power of linear time’, and the latent desire within companies and markets to ‘resolve tensions among priorities, loyalties and accountabilities’ (Greenhouse 1989, p. 1638). ‘Cetology’ plays, instead, on temporal mutinies and dis-containments, monumentalising generations of history and tradition (the aura of the whale, the skills employed for re-fabricating the plastic) and the social, economic, cultural, and biological entanglements that mount, after time (and after industry, mass production, economic globalisation, et cetera).

The work also transforms the proximity that visitors might experience in relation to the furniture, from chairs that command the patio and BBQ to artefacts that hang, distant, in the air of the natural history museum, *as if* they might perform additional normative and interpretive functions. The plastic commodities-*cum*-whale take on a different kind of ‘aura’ in this situation, perhaps which they lost in mass manufacture and consumption. Importantly, though, the chairs acquire this aura, or renewed capacity for ‘voice’, as geological agents and as *ruin* (rather than as industrial innovations or wealth generators). This negative extensiveness is juridically important, where it happens at intra-temporal and cumulative proportions, impacting a community’s experience of *harms,* if not the most basic physical processes of the planet. It relates, in fact, to intensively-scaled or ‘new’ harms, defined by philosopher Judith Lichtenberg (2010) as the intended and unintended consequences of actions, which stretch across—and thereby uncontain—a multiplicity of time-scales, networks, actors, actions, and affects. The new harms relate, in Lichtenberg’s analysis, to diffuse social processes, which gather their momentum and impactfulness over time, and after stable public policy development and millions of actions that proceed according to legal rules and accepted practices. Such harms are widely identified by legal philosophers as out-sized and irregular, and as struggling to attain moral constraints (positive and negative duties to avoid aggregate harms) or effective redress and recognition within modern legal systems (Lichtenberg 2010, Young 2013, Morton 2013). Specifically, Lichtenberg clarifies, ‘[T]he model of harm underlying the classic formulation of the harm principle—discrete, individual actions with observable and measurable consequences for particular individuals—no longer suffices to explain the ways our behavior impinges on the interests of other people’ (Lichtenberg 2010, p. 558). It also does not, as the Anthropocene thesis suggests, account for the impact on non-human existences (biotic, abiotic) and communities.

‘Cetology’ is important, in sum and as a starting point for the coming analyses of corporate legal order, because it probes the gallery visitors’ attentiveness to natural-cultural entanglement and colliding earth and world histories and time-scales*.* Commodity and natural histories hang in the air of the museum, riveted together and ominous, ‘the non-mortal remains of mortal beings’ (Brown 2015 p. 209). Jungen’s sculpture references shifting shapes of harm and injustice, as it invites visitors to contemplate how material remnants of the way that we live and, sometimes, dominate others might be *concretely* understood in a monumental allegory of plastic-driven extinctions.

## II

A material kind of distributed impactfulness, and even ruin, has long been associated with commercial organisations and industrialisation (Polanyi 2001). Legal debates that discuss and assess modern-day corporate governance and Corporate Social Responsibility (CSR) reflect this, and identify the corporation as an influential and impactful social actor since the inter-war economy (1918–1939). Corporate power augmented in this period and throughout the twentieth century as a factor of size, growing operational capacity, and the integration of corporate units within multi- and transnational firms (Parkinson 1995). This process accommodated, and was in fact advanced by, the rising interests and expectations of investors (at the end of the nineteenth century) and, later on, by investment managers (late twentieth century), seeking ‘profitable outlets’ for capital and trading, after the institutionalisation of (their own) limited liabilities (Ireland 2017).^10^

Scholars working across different disciplines (law, anthropology, sociology) observe how migrations of force and affect attend the global networks of companies, including flows of labour, resources, and information across human, plant, mineral, animal bodies and environmental change processes (Fortun 2009; Dolan and Rajak 2016). Impact and sometimes harm is cultivated across the complex systems of production, including decentralised structures of enterprise association and direct and indirect forms of political engagement by companies, which grow companies’ ‘influence over broader relationships’ and over the ‘institutions that shape and constrain the substantive realisation of human rights’ (Macdonald 2011, p. 549). Relevant harms, which gestate across the actions, organisations, and supply chains involving companies, commonly bypass domestic accountability traditions (in tort law, criminal law, environmental controls, labour law and health and safety legislation), due to the transnationalisation of corporate activity and the diffusion of harms (Macdonald 2011; Young 2013). Alternatively, corporate impacts do not attract legal enforcement in countries seeking to improve their competitiveness, or to attract investment, by reducing the costs of social and environmental compliance and mitigation for companies at law (Simons and Macklin 2014; Banerjee 2012; Baxi 2016).

Voluntary-styled corporate social responsibility (CSR) and corporate environmental responsibility (CER) programmes have rapidly advanced, in this normatively and materially fragmented context, as a practical means of ‘enlightening’ business actors about their impacts and associations, with near and distant communities (Campbell et al. 2007). In legal terms, these programmes rely on reflexive and soft law, and *mainly* comprise disclosure laws, designed to encourage corporate reporting on non-economic performance and environmental, social and governance (ESG) factors ‘material’ to businesses;^11^ directors’ duties concerned with managers’ regard for ‘wider’ interests, including labour, suppliers, customers, communities and the environment, when promoting the success of the company for the benefit of the members;^12^ and non-binding codes and international instruments that aim to improve reflexivity at companies about impacts, including distant ties and effects, and ‘respect’ for human rights.^13^

Many corporate legal scholars express concerns about the adequacy of this approach. Their reluctance mainly concerns the predominance of soft law and guidance to companies, the absence of enforcement mechanisms and standards, and the maintenance of market institutions that seem to militate against the advance of non-economic priorities. Companies must still proceed with CSR and CER on a ‘business case’ basis.^14^ Shareholders and financial markets still retain legal priority over other stakeholders and communities.^15^ Company lawyers and policy makers refer, in response, to the engagement that is now being demanded of companies with matter and materiality. This turn (to matter) is evident in laws that direct companies to account for ‘environmental, social, and governance’ *risks* and *factors*, to institute non-financial *performance* indicators and resource *efficiency*, and to consider ‘the responsibility of businesses for their *impacts* on society’ (European Commission 2011), whilst ‘*contributing to society*’ and ‘promoting *long-term sustainable* success’.^16^ Companies are invited to perform risk-based ‘due diligence’ on actions and associations, to dig deeper into their subcontractors’ business practices, and to account for the material and ‘multi-dimensional nature of CSR’—as ‘at least’ covering community and stakeholder involvement, human rights, labour and employment practices, tax and environmental issues, and combating bribery and corruption (European Commission 2011, at 3.3). The overlying demand, as such, is for a grounding of companies in consequential matters and relationships, perhaps a wholehearted coming *to terms with the materiality of itself.*

Yet, a dissonance or countermand is also at play. This dissonance arises where the recent history of corporate law and governance is not really material or grounded in matter (never mind geology). It is, rather, better understood as part and parcel of the idealistic and modern reflexive tradition, which has expressed itself powerfully within the economic sphere through the recognition and extension of separate corporate legal personality, as the ‘systemically privileged juridical “human” subject’ (Grear 2015, p. 227; see also Baars 2019, a ‘legal concept to “congeal” relations of production’, p. 11). This idealistic history of corporate legal personhood is concerned less with the build-up of matter and consequences around profit and investment as it is concerned with the advance of the corporate form and *expectation*, and with the development of industrial reason as a means of reconciling different bodies, interests, and goals. It probes, in policy terms, ‘what is expected of enterprises’ (European Union Commission 2011, at 1.2), and settled rights, interests, and purposes for companies, specifically entrepreneurial applications and the purposes of wealth generation.^17^

Evidence of this history of expectation can be gleaned from classic Anglo-American company law and governance debates from the start of the twentieth century, which, as Lyman Johnson (2017) argues, elaborated on the internal governance systems and structures of the company and swept away older debates about the ‘power’ and ‘public’ obligations of corporate entities. US law professors Merrick Dodd and Adolf Berle, Lyman points out, used their leading intervention in the 1930s to direct attention to the ‘interests of the company’ and to the duties of managers, as guardians of the corporate body and, also, of wider interests, entangled with business. So that ‘[R]ather than addressing the obligations of the business itself’, and the material nature of corporate organisations and activities, the classic debates ‘emphasized the duties of those who governed the business’, after ‘abandoning a regulatory philosophy’ from around the end of the nineteenth century (ibid. p. 571). The distinction of expectation and management here is important, where it suggests that the common reading of the Berle-Dodd debate, as between stakeholder versus shareholder characterisations of corporate actors, is less than the full story. Equally important, for this analysis, is the foundation that this classic company law debate provides for *avoiding* corporate matters and consequences *as a whole*, whilst conceptualising the company in terms of (only) contractual metaphors and fiduciary duties, managerial norms and calculations (rather than as an institution with a seperate and material existence in its own right). This dematerialised reading or ‘interpretation’ of the company matters where it makes tracing the social and environmental imprint of companies difficult, since much of the disclosure and veracity required continues to lie with managers (as guardians). It has tended, in practice, to also undermine common participation within the company and to limit managerial discretion to the shareholder primacy norm, even against pressure for change, where this offers a strong means for ‘clarifying expectations’ and for improving market discipline over directors (entrenching, as such, a much-criticised theorisation of companies by financial economists from the 1990s).^18^

Recent UK reforms within corporate law and governance point to growing unease with the ongoing consolidation of *expectation,* over (attention to) *matter* (which Johnson describes as ‘corporate deregulation’ in the CSR and CER context). Policy makers try to counter this unease as above, encouraging more material concerns and modes of reflection at companies (and investors). But this work is not easy, or altogether coherent, when expectation and industrial reason combine to make matter ‘amenable’ (to management), and the main mode of communicating with companies and their decision-makers in the deregulated sphere is still the market and demand—arenas built for *immediacy*, *instrumentalism*, and *economic* ambitions (i.e. competition, market discipline, corporate self-regulation), and vulnerable to inequalities in voice and ‘reified’ wills (Teubner 2020). The problems are visibly borne out in recent research (Lawrence et al. 2020), which suggests that UK companies (at least) are still highly shareholder-orientated, in practice and long after the development of CSR and CER systems for the regard of intergenerational matter, including environmental crisis and inequality.^19^ Dividend payments from FTSE 100 companies hit a record £110.5 billion in 2019 in the UK—a rise of 10.7% over 2018 and more than double the £54 billion paid out in 2009 (Lawrence et al. 2020, p. 2). Guidance for companies introduced in response to the COVID-19 pandemic has not (to date) advanced legal or substantive changes to mainstream corporate expectations and priorities, which still, too often, leave existences confined to the margins, to ‘ruin on’.^20^

## III

In his 1934 essay on Franz Kafka, Benjamin talks about the author’s interest in exposing the ‘fullness of the world’, as ‘the only reality’ (Benjamin 2015, p. 127). ‘Everything forgotten mingles’, Benjamin says of Kafka’s atmospheric literature, ‘with what has been forgotten of the prehistoric world, forms countless, uncertain, changing compounds, yielding a constant flow of new, strange products’ (ibid.). Kafka’s animation of different forms and non-humans interests Benjamin, where it meets his understanding of the task of the writer, which is to move ‘cosmic ages in his writings’ and to do so ‘in the presence of others: He does not live for the sake of his own life, he does not think for the sake of his own thought. He feels as though he were living and thinking under the constraint of a family’ (composed of ‘human beings and animals’) (ibid. p. 126). By following the families’ bidding, Kafka moves ‘the mass of historical happenings as Sisyphus rolled the stone’ (ibid. p. 126). The world’s ‘nether side comes to light; it is not a pleasant sight’, says Benjamin, but ‘Kafka is capable of bearing it’ (ibid. p. 126), as he writes and exposes ‘insoluble behavioural problems’ and the ‘limits of understanding at every turn’ (ibid. p. 120).

Things are routinely forgotten by Kafka’s characters in Benjamin’s reading of the main works; (of *The Trial*) ‘It is as though nothing new was being imparted, as though the hero was just being subtly invited to recall something that he had forgotten’ (Benjamin 2015, p. 127). This forgetting does not mean that certain happenings ‘do not extend into the present’: on the contrary, some things are ‘actual by virtue of this very oblivion. An experience deeper than that of an average person can make contact with it’ (ibid. p. 126). A ‘prehistoric world’ begotten by ‘guilt’ (ibid. p. 128) and arenas like ‘attics’, the place of collectible but ‘discarded, forgotten objects’ (ibid. p. 129), are drawn, by Benjamin, alongside Kafka’s fascination with forums of the legal process. This is where ‘the necessity to appear before a court of justice gives rise to a feeling similar to that with which one approaches trunks in the attic, which have been locked up for years’ (ibid. p. 129). Benjamin is intrigued by Kafka’s unorthodox method for attending the discounted and, also, by related questions of justice. He highlights Kafka’s deployment of non-humans as ‘receptacles of the forgotten’ (ibid. p. 128) and as ‘outsiders to the law’. ‘This much is certain’, he observes, ‘of all of Kafka’s creatures, the animals have the greatest opportunity for reflection. What corruption is in the law, anxiety is in their thinking’ (ibid. p. 128). Kafka’s animals are possessed, he says, with ‘the natural prayer of the soul’, which is ‘attentiveness’ (2015, p. 130), in a situation where ‘the gate to justice is learning’ (ibid. p. 135).

Benjamin developed these ideas—about the fullness of the world, oblivion amongst purportedly ‘rational’ actors, and the limits of human understanding, including *at law*—in a number of his works in the 1920s and 1930s. He addressed themes that Jungen also invoked for us, including the creaturely (as reflexive), the ability of the non-human (the chair, whale) to offer historical testimony, and an ironic shattering of traditions amidst otherness, ‘which is the obverse of the crisis and the renewal of mankind’ (ibid. p. 215). Benjamin offered an early (1925) systematisation of these ideas in his work about the mourning play, *The Origin of German Tragic Drama* (Benjamin 1998). This is where, in the ‘Prologue’ to the main text, Benjamin introduces the concept of ‘natural history’ [*Naturgeschichte*] as a horizon expressive of social historical content, and as destination for the material or textual ‘fragment’ that lives on beyond ‘intentionality’ and the philosophy of consciousness. In later works, which study industrial culture and the ‘debris’ of the commercial arcades of Paris in the nineteenth century (1999), Benjamin documents occurrences that live on, more specifically, beyond the accommodations (and victories) of modern commercial systems and ideologies, including their value-structures.

Benjamin, like the present paper and issue, was keen in these works to move away from modern and idealistic conceptions of societal-nature relations, which categorised nature as pre-given and as the back-drop to human action (and instrumentalism). He emphasised, instead, the entangled and historical nature of being (origin ‘describes that which emerges from the process of becoming and disappearance, … an eddy in the stream of becoming’), and studied occurrences beset by ‘the past and the subsequent history’ of their essences (Benjamin 1998, p. 47). He deployed concepts like ‘transience’ and ‘decay’ as a means of capturing matters of consequence, fragmentation, ruin, and living on; and as horizons of existence that could be understood *secularly*—that is, as not exclusively anthropogenic in their development (not relatable only to the concerns of humans). Benjamin saw in this living on proof of the fullness of the world, but also, the historical dynamization of intransient ideas and calculations (the social and material content of the idea). He used the insight to suggest that we might trace living history from the *surfaces* of ‘surviving’ objects, working from visual concreteness and bodies of affect that reach into the realm of experience, and *demand translation as such*. These remnants or bodies demand interpretation, as a matter of justice, where they undercut the usual antinomies between causality and morality, natural history and world history (invoking the juridical problem identified by Lichtenberg 2010; Hanssen 2000, p. 36). But also because the decaying matters presented or contained, for Benjamin, new possibilities for reading history *against the grain* of what humanistic actors had valued or accounted for (so far).

To this end (of justice), Benjamin developed a citation mode of ‘writing’ social history. It sought to document ruin, decay, and discontinuity, carving out dates for their ‘physiognomy’ (Benjamin 1999: N11). The philosopher pioneered creative techniques of montage and juxtaposition, collecting objects, texts, and experiences, which could evoke different times and matters in the context of loss, and create ‘flashes of recognition’ (1999, N1,1; 2015, at p. 247: ‘the past can be seized only as an image which flashed up at the instant when it can be recognized’). Benjamin’s works commended the techniques of allegory, aura, juxtaposition and distance, for adding to the comprehension of ‘the social bases of the contemporary decay’ (Benjamin 2015, p. 216), and for adopting a ‘dual insight’.^21^ As allegorist, Benjamin’s theories sought not to affirm the ‘truth’ of this nether world, nor to restore any ‘truer’ set of associations ‘that may have been lost or obscured’ (theorist Craig Owens on the ‘allegorical impulse’ 1980, p. 69). His historical method invoked, rather, multiplicity and transience as really-existing contemplative objects, *adding* layers of meaning, writing *over* commodity histories with effects and experiences, to enact a ‘vertical or paradigmatic reading of the correspondences on a horizontal or syntagmatic chain of events’ (Owens 1980, p. 73). Benjamin’s philosophy aimed, as such, at a redemption of the semantic potential of the past, contextualizing objects and experiences in such a way that their philosophical-political meaning might be revealed, and perhaps also ‘counter-posed’ as a site of *contingency*, *consciousness* and *duty.* ‘It is … of decisive importance’, he said, ‘that a new partition be applied to this initially excluded, negative component so that … a positive element emerges in it too’ (Benjamin 1999, N1a3).

## IV

This paper started with ‘Cetology’s’ illumination of ruin and the impacts that accumulate after commodities and industries in uncontained times. It examined corporate entanglements and responsibilities, which, also, intersect with large-scale environmental crises and legions of the impacted. Help with this task was teased from Benjamin and Adorno on the ‘idea of natural history’, and the deployment of ruin and allegory as a means of returning actors and systems to the ‘fullness’ of their pasts. The paper, in this final part, explores how shifting shapes of harm and injustice that are relatable to business actors might be sensed and interpreted after this art and text and their methods, within legal and responsibility systems now facing alterity, distress, and ruin. It contemplates how the company might be seen, secularised and made dutiful towards diffuse impacts, not through more management and governance (strategies critiqued in Part II) but after re-contextualising the company among matters and the ‘historical imagination’.

The prospect sounds imaginative. It does accord, though, with growing amounts of legal and normative engagement with diffuse matters and the ‘age of consequences’ at law. Such engagement is evident, for instance, in the recent increases in climate litigation, which seeks to hold governments and private organisations responsible for climate change-related damage and/or threats of damage.^22^ Legal scholars and practitioners, discussing the cases, highlight the prospects for large-scale harm objects and social-natural entanglements to be ‘legally disruptive’ (Fisher et al. 2017). This disruption occurs where certain diffuse, uncertain, polycentric, spatially and temporally overflowing matters, which enter experience, ‘promote reflection’ about the nature of legal adjudication and legal reasoning embedded in the law (ibid. p. 260). Courts and lawyers, confronted with rising matters and temporal mutiny, can work in imaginative and activist ways to expand the capacity of law to hold public and private entities accountable for material damage and threats, developing and extending (for instance) domestic precedents on twentieth-century mass harms (e.g. tobacco and asbestos litigation, business and human rights) (Aristova 2018, Rühmkorf 2015, Ganguly et al. 2018). Major advances in scientific knowledge about harm and environmental transformation help to ‘strengthen the causal link’ between protagonists and damage (Ganguly et al 2018, pp. 10–13), and to afford a broader range of arguments and litigation strategies to lawyers (Fisher et al. 2017; Mason-Case 2019). Legal scholars researching in this vein draw, too, from theories and methods of the ‘new materialist’ scholars’, as a set of ideas comparably attentive to the relational constitution of consequential matter, and to the disruptive potential of trans-generational ‘collectives’  and ‘raw materials made of poor humans and humble non-humans’ (Latour 1993, pp. 114–115; Haraway 2016; Davies 2017).^23^

But what of the corporation in this matter-bound context, and the opportunities that lie around it for legally and normatively ‘disrupting’ company law and the political economies of ‘business as usual’, including shareholder primacy and disembodied corporate governance? In Part II, the paper identified corporate law (and CSR and CER) as an area where rising matters are made ‘amenable’ to expectation, and must currently achieve economic status and recognition, or they might fall into the tide of ‘natural history’—of ‘things created by man, yet lost to him’ after ‘progress’ (Adorno 1984, p. 117). Instructive, on this point, is the recent US decision, *New York v Exxon Mobil* (2019),^24^ part of the second wave of climate litigation (as characterised by Ganguly et al 2018).^25^ In this case, the global energy corporation, Exxon Mobil, successfully defended itself against allegations of fraud for potentially misleading investors about climate risks to the company. The New York Attorney General, bringing the case, failed to establish that the corporation made material misstatements or omissions that could have misled any ‘reasonable investor’ about its practices for accounting for climate risk. The court came to this conclusion after reviewing the non-financial information issued by the company,^26^ but also by gathering evidence about the ‘materiality’ of the information for managers and investors. The court found ‘no evidence’ that ExxonMobil’s non-financial information had ‘any market impact at the time they were published or that *investment analysts* took note of the contents of these documents, which were widely disseminated’ (*New York v Exxon Mobil* 2019, p. 8, emphasis added). The court accepted the ‘total mix of information’ available and, also, evidence about the satisfaction of investors with information addressed to shareholders on the management of climate risk.^27^ ‘The Office of the Attorney General offered *no testimony from any investor* who claims to have been misled’, summarised the court (ibid. at p. 38, emphasis added), adding that ‘No reasonable investor during the period from 2013 to 2016 would make investment decisions based on speculative assumptions of costs that may be incurred 20+ or 30+ years in the future’ (ibid. at p. 34).

This summons of *investors*’ capacity for judgement and *better nature* is echoed across mainstream corporate governance and law. Relatable court judgements in the UK and beyond confirm ‘business judgement’ as a first-order forum for reflection on large-scale, inter-temporal risks and phenomena. Courts remain reluctant to assert obligations that could counteract or interfere with commercial expectations. They affirm moral rather than legal obligations at the international level to mitigate climate change risks at companies (in *Re. Greenpeace Southeast Asia and Others* 2019),^28^ and enforceable domestic obligations for directors and companies to meet commercial expectations, sometimes over ‘green’ hopes and aspirations (in the UK, *R (People and Planet) v HM Treasury* 2009).^29^ The courts, in these cases, would seem to pull back from the ‘disturbed certainties’ that attend the integration of geological and human time-scales, and the possibilities that lie, within this, for ‘temporally dis-contained’ or ‘vertical’ norm contemplation. Legacies of affect must still find their place among ‘competitive principles’ (cited as a legitimate limit on ‘materiality’ in *New York v ExxonMobil* 2019, at p. 35), as markets remain predominant for collating public preferences and guiding ‘judgement’ about harm objects (evidence is led about the priority of ‘cash flow’ for informing expectations at ibid, p. 39). The justices miss the widening cognitive and legitimacy-crisis, which now attends modern competitive economic systems, struggling to comprehend how nature and history ‘break apart and interweave’ in their company, or to develop different principles of ‘care’ and ‘attention’ for the hazards and losses left behind.

An alternative approach and method is probed by this paper, which wants to expand the ways in which corporate lives are talked about, regulated and experienced, away from the normalised conceptualisation of companies as self-knowing and self-governing actors. This method engages the ‘idea of natural history’ as a register for re-thinking corporate impact and entanglement as a seperate and material existence, which bears upon issues of justice and equality, and seeks to collate wider experiences apart or distinct from the company and investors: to ‘extend the list of movements that have to be taken into account’, as Bruno Latour summarises for the new materialists, also intent on ‘tracing’ the affect that attends the march of economization and modern frailties (Latour 2018, p. 87).^30^ Importantly, the ‘more that we see the erosion of the constitutive character of the mind’, of corporate management ‘adumbrated’ by loss and crisis, ‘the more insistent becomes the need not just to register existing reality, *but to reflect upon it and understand it*’ (Adorno 2006, p. 127, emphasis added). The *matters*, which extend beyond law and order (and the proclaimed ‘end’ of corporate legal history), speak in this proposition not against certainty and expectation (as the judges and indeed shareholder primacy advocates fear), but to ‘the ruin of time, and a process of decay that is at the same time a process of crystallization’ (Arendt, in Benjamin 2015, p. 55). This ‘process’ (time + ruin+contemplation) is important and distinct, where it enacts presence, participation, and imagination from and for the margins of economy and law as a prospect emboldened by time (the ruin as ‘second nature’ (Adorno 1984), ‘interpretation presupposes the decay of systems’ (Adorno 2006, p. 127)). Critically, it (time) assigns *contingency* to modern structures and actors, including the corporation, relating consciousness and duty to the ‘ruining’ and ‘lost’ as well as the participant and lively, and finding witnesses among the mutinous and creaturely for the ‘anxieties’ of modernity’s ‘god tricks’, including industrial reason.

Jungen, Benjamin and Kafka deploy the historical imagination and allegory—the ‘intuition of impermanence’^31^—as a technique for extending presence, after this spirit. Hard though this idea is to grasp, from a legal perspective, the intuition that drives it—and art like ‘Cetology’—is fitting to the fullness that attends corporate actions, and the entanglement with ‘new’ harms. In these full or swampy situations, which throw up co-existence and temporal mutiny at every turn, corporate responsibility scholars, turned ‘allegorist’, might also want to *juxtapose* companies’ affect histories to the moral captions that companies generate (industrial reason’s ‘utopian potential’). They might relate intergenerational losses after new forms, which try to ‘make explicit’ the historical nature of what has been forgotten or ‘naturalised’ (as losses, as beings impacted), learning from the ‘surfaces’ of surviving objects, ties, and communities in geologic and montaged forms.^32^ Such methods are not meant to be a way to make the pile-ups of affect more ‘true’ for the law than, say, the economic achievements of companies and markets, as readers of Benjamin may anticipate (‘to article the past historically does not mean to recognize it “the way it really was”’ Benjamin 2015, p. 247; ‘allegory could not exist if truth were accessible’, Cowan 1981, p. 114). Adopting a ‘dual insight’ (Benjamin), the law after the historical imagination *cross-checks* calculation, and eternal trust in the corporate form, with a new-found receptiveness to the build-up of matter and histories of harm. It brings mortified and estranged natures ‘out of infinite distance’ and ‘into infinite closeness’, toward interpretation and the study of ‘decay’ as well as ‘detail’ in transient forms.^33^ It becomes capable of ‘bearing’ nether sides, and attics, and of counter-posing affect and experience to commercial expectations and priorities, expanding (thus) the pathways for countenancing ‘business as usual’ with new forms of historical reflection about corporate entanglement and adding to duty or obligation at companies for legacy matters.^34^

Part IV offers, with this, to clarify the paper’s interest in substituting the naturalness of the company, and management, with spontaneous concern for the affected and for existences that lie within the horizons of ruin and disorder, at law. Times’ uncontainment, in light of the crises the world is facing, could and should invoke a change in expectation for the company law field, assigning contingency to the corporation (and economics) and provoking a new mode of reflection about corporate entanglement and responsibility at law, and across domestic and international legal fields.^35^ The contribution that this essay and its interlocutors wants to make is to highlight and to explain that an important part of the justification for this turn towards obligation lies with matter and geology, animals and attics, rather than with the extension of idealistic and economic thinking or incentives. It is where meeting the challenges of the epoch requires a renewed commitment to ‘earthing’ but, also, *‘de-centering’* the corporation from its default position of judgement and self-knowing, matter by matter, community by community. Before things reach the air of the natural history museum, perhaps.

## Conclusion

This paper has talked about art, theory and the ‘idea of natural history’, as a means of thinking corporate entanglement and responsibility for the Anthropocene. The findings invite lawyers, policy makers and scholars to increase their level of attentiveness to corporate matters, and to attend the worlds, existents, environments, and autonomies impacted by business organisations over time. The primacy that the interlocutors (Jungen, Adorno, Benjamin, Kafka) accord natural history is powerful, in this context, in its capacity to position corporate organisations *within* natural-cultural entanglement, as neither ‘bad guy’ (i.e. as the persistent subject of scandalisation) nor ‘saviour’ (as meeting all demands for responsiveness). Companies become, instead, *implicated* in, and bearing responsibility for, the kind of ruin that the epoch of the Anthropocene is making concrete and unwise to (only) bound, divide up, calculate, and surpass. This modest end-point to the analysis captures the subtle radicalism of the artists and writers studied. It also, and finally, reveals what it means to the author to ‘de-center’ the corporation, and to release earthly existences from the usualness of submitting to corporate expectation, one gulp of fresh air at a time.

## Footnotes

1 The term ‘Anthropocene’ refers to a new proposed geological epoch in which humans and human societies have become the dominant influence over climate and the environment. On the definition and implications, see Steffen et al. (2007). Relating a ‘New Climatic Regime’ and the affect histories of recent modernity and economic globalisation (dated to eras of deregulation and the dismantling of the welfare state in the 1980s and 1990s), see Latour (2018, pp. 17–21).

2 Relevant periods of industrialisation are commonly dated by Anthropocene scholars to around the 1800s, to the industrial revolution, see Malm (2016) and/or to a later ‘accelerated’ period of post-war economic development, see Steffen et al. (2007) and also Chakrabarty (2018, pp. 11–13). Latour (2018, p. 10) names relevant affect migrations as ‘without form and nation’, and as concerning ‘climate, erosion, pollution, resource depletion, habitat destruction’.

3 The term ‘corporate legal order’ is used in this paper to reference a body of laws and institutions that undergird the elevated position of companies, particularly large and listed multinational companies, within the global economic order as a whole. It comprises, at least, corporate and financial law, attendant regulatory and governance institutions, as well as property law and private law.

4 ‘Matters’ relatable to the company are referenced throughout the paper (as ‘corporate matters’) and are causally linked within this to the latter’s actions, mediations, and networks. In line with scholarship of law and ‘materiality’, these matters also presume the agencies of living organisms and ‘vital matters’, and the force associated with aggregate existence, i.e. geological change. For a discussion of the terms, and on how law comes to care about such ‘matters’, see Kang and Kendall (2019) and Davies (2017).

5 Jungen’s sculpture precedes much recent discussion about and popularisation of the Anthropocene concept, and carries, as such, a telling or predictive power.

6 ‘Fortleben’ translates as ‘continuing life’ and ‘living on but also living away’ (Tomlins 2018, p. 7). The concept is drawn by Tomlins from Walter Benjamin’s work on translation and (acting beyond) intention, for instance ‘The Task of the Translator’ in Benjamin (2015, pp. 70–82).

7 Numerous websites publicising the sculpture and Jungen’s art relate this detail (of price tags being still visible in some parts of the sculpture), for instance, see Orozco (2012): https://justement.org/post/15403221505/brian-jungen-shapeshifterpartial-cetology-126 (Accessed 25 August 2020).

8 The term ‘ur-historical’ relates to the work of Walter Benjamin (Benjamin 1999) and his development of ‘the idea of natural history’ as an ‘ur-history’ of the nineteenth century that sought to ‘trace’ living history from the surfaces of ‘surviving’ objects and ‘ruin’, and by working with and from visual ‘concreteness’. For a discussion, see Buck-Morss (1991), Chapter 3 and Chapter 6.

9 ‘Whenever an historical element appears it refers back to the natural element that passes away within it’ (Adorno 1984, p. 120).

10 For more on the history of limited liability and the (later) emergence of corporate ‘separate legal personality’, Ireland et al. (1987).

11 In the UK, see Sections 414A-D Companies Act 2006 (c.46) (implementing Directive 2014/95/EU) and the Modern Slavery Act 2015, Section 54. For a summary of obligations for environmental reporting, see HM Government (2019).

12 Section 172 Companies Act 2006 (c.46). On the attention demanded of shareholders, see the UK Stewardship Code 2020 and the EU Commission ‘Action Plan on Sustaining Sustainable Growth’ (2018), available for download at: https://ec.europa.eu/info/publications/sustainable-finance-renewed-strategy_en. Accessed 20 August 2020.

13 Global instruments include the United Nations Guiding Principles on Business and Human Rights (UNGP) (www.ohchr.org), the United Nations Sustainable Development Goals (UN SDGs) (https://sdgs.un.org/goals), and the OECD Guidelines for Multinational Enterprises (https://mneguidelines.oecd.org/). Accessed 20 August 2020.

14 Legal advice on the level of directors’ ‘regard’ towards non-economic stakeholders and interests is set out in *R (on the application of People and Planet) v HM Treasury* [2009] EWHC 3020 (Admin) Queen’s Bench Division (QBD), with the limits of the ‘business case for CSR’ confirmed at paragraph 12, and discussed in Talbot (Talbot 2013).

15 Talbot (2016), Baxi (2016), Villiers (2018).

16 UK Corporate Governance Code 2018, Principle A.

17 Grear 2015; see also on the legal history of corporate ‘separate legal personality’, Ireland et al. (1987).

18 Related by Johnson (2017), Ireland (2017), and in detail by Talbot (2013, pp. 102–144).

19 Discussing and critiquing the environmental crisis and situation at law for corporate lawyers, Sjåfjell (2018), Talbot (2016). Discussing the continued advance of competitive and growth-orientated economic institutions, and their limited cognitions of matter (including environmental crisis and inequality), Hickel (2019) and Report of the Special Rapporteur on extreme poverty and human rights (Philip Alston) (2020).

20 Recent advice is set out in Financial Reporting Council (2020), available at: https://www.frc.org.uk/about-the-frc/covid-19/company-guidance-updated-20may-2020-(covid-19) (Accessed 25 August 2020). Sjåfjell (2018) makes the case for amending the Companies Act 2006 (CA 2006) to clearly advance sustainability and stakeholder purposes (over shareholders), assessing also scope to develop these purposes within the *existing* CA 2006 Section 172. Talbot (2013) discusses difficulties with the present legislation, probing the point about expectations, capital markets, and investors.

21 Of this realm, he said, ‘Its rhythm is apparent only through dual insight. On the one hand it needs to be recognized as a process of restoration and reestablishment, but, on the other hand, and precisely because of this, as something imperfect and incomplete’ (Benjamin 1998, p. 45).

22 Recent corporate litigation includes the 2015 NGO petition seeking to determine the accountability of ‘Carbon Majors’ for the ‘human rights implications of climate change and ocean acidification’ at the Philippines Human Rights Commission (*Re. Greenpeace Southeast Asia and Others v Carbon Majors* (2019), Case No CHR-NI-2016–0001 (2015), judgement at https://climatecasechart.com/non-us-case/in-re-greenpeace-southeast-asia-et-al/ (accessed 20 August 2020)); and *Milieudefensie *et al. *v Shell plc*, currently before the Netherlands domestic courts, with human rights and tort obligations in contention, which, importantly, seek to extend the outcome of *Urgenda v Dutch State* (2019, Sup. Ct. of the Netherlands, Number 19/00,135) to business organisations. See the legal summons, at https://www.foei.org/wp-content/uploads/2019/04/2019-04-05-SUMMONS-dagvaarding-unofficial-translation-of-the-Dutch-original.pdf (accessed 20 August 2020).

23 Davies describes and defines new materialism as broadly situating ‘human’ action in a ‘material world where all matter, living and non-living, is related, where objects have their own vitality and resistance, and where agency emerges in relation rather than as an existing quality’ (2017, p. 66).

24 *New York v Exxon Mobil* (2019) N.Y. Supt. Ct. 2019, Doc. No. 567.

25 Earlier waves of tort claims against ExxonMobil, which did not establish sufficient proximity or causal links to large-scale atmospheric change for success, are set out by Ganguly et al (2018). ‘Specifically, the court concluded that the plaintiffs could not establish causation because there was “no realistic possibility of tracing any particular alleged effect of global warming to any particular emissions by any specific person, entity, [or] group at any particular point in time”’ (2018, p. 9).

26 The relevant ‘information available to ExxonMobil investors during the relevant period included an annual, publicly-filed report called the Outlook/or Energy, the two March 2014 Reports, ExxonMobil’s Form 10-Ks, ExxonMobil’s annual Corporate Citizenship Reports, and a host of other publicly available information that was not the subject of testimony at trial (including ExxonMobil’s Annual Shareholder reports)’ (2018, pp. 9–10).

27 The Christopher Reynolds Foundation and Arjuna Capital ultimately agreed to withdraw their proposed shareholder resolutions (inviting directors to improve reporting on climate change risk and management) based on information shared by the company at the December 2013 meeting and ExxonMobil’s undertaking there to address climate related inquiries (2018, pp. 14–15).

28 See note 27.

29 Queen’s Bench Division, Administrative Court. In this English case, the Court confirms matching legal obligations in the UK Companies Act 2006, Section 172, for directors and investors not to ‘skew’ the performance of companies ‘in an anti-competitive way’ (at paragraph 12). See also, recent EU developments, concerned with shareholder and investor manager duties to attend ESG and the ‘long-term’ but within (still) growth-orientated models of economic development, discussed in Möslein and Sørensen (2018).

30 In his classic text, *We Have Never Been Modern* (1993), Latour also engages the ‘idea of natural history’ (though he does not use this term). Deficits in meaning and existence arise in his theory after modern constitutionalism and scientific reason partition some ‘actors’ or ‘actants’ into the ‘background’ in the search for ‘a society with predictable and stable interests and stakes’ (Latour 1993, pp. 10–11). ‘Discounted’ elements do not disappear or become permanently disassociated from modern systems and organisations, for Latour either. Rather, natural-cultural or ‘hybrid’ phenomena ‘live on’, and form part of our experiences (see, for instance, pp. 1–3). Latour’s wider work suggests that networked and mediated matter might be ‘traced’ on a materialist horizon, when we ‘study in detail the world of production of hybrids and the world of elimination of these same hybrids’ (and discover that ‘we have never been modern’, Latour 1993, pp. 46–47). The present reading of Adorno (1984) and Benjamin (1998) is intended to add to, but also *query*, the new materialist empirical method and promise, in the development of allegory and accounting for ‘ruin’, the ‘lost’, and ‘decay’. See Fortun (2014).

31 For more on allegory, see Owens (1980), Cowan (1981), and Buck-Morss (1991, pp. 160–201).

32 See Hofstätter (2019) on Adorno’s admiration for Beethoven’s ‘late’ style as an evocation of this explicitness and the natural history critique. Haraway (2016) also talks in a relatable way about working with ‘surviving’ natures and communities, pursuing engagements that cultivate the ‘lives’ and the ‘details’ of ‘multi-scalar, multi-temporal, multi-material worlding’, advanced by ‘corporations, farms, clinics, labs, homes, sciences, technologies, and multispecies lives’ (p. 115), clarifying ‘details’ and telling ‘stories’ that could ‘link actual beings to actual response-abilities’ (p. 116).

33 Teubner (2020) uses the term ‘*mediation*’ in a relatable context (of presenting matters and ‘preconceptual affect’); Latour (1993, 2018), for long now, has talked about ‘*diplomacy*’. The reference here to ‘dual insight’ and *‘*cross-checking’ is informed by Benjamin and his advice about transience (Benjamin 1998, p. 45, see note 26) and not allowing ‘conformism’ to overpower matter (2015, p. 247, in ‘Theses on the Philosophy of History’).

34 Teubner (2020) talks about ‘counter-rights’ in this context and the potential for a ‘new law’. Tomlins identifies the promise of law adopting a different (live, unsocialised) relational other, such as history, the past, or (more than human) justice (Tomlins 2007). Haraway (2016) relates, in some detail again, the materialist’s efforts to command ‘details’ and tell ‘stories’ that ‘link actual beings to actual response-abilities’, where revisiting the state of matter in concrete (ethnographic, sociological, artistic, experimental) terms means ‘a muscle critical for caring about flourishing gets some aerobic exercise’ (p. 116).

35 Scholars of the ‘new corporate accountability’ have widely identified relevant fields of obligation, for instance in contract law, tort law, labour law, international human rights and environmental law. Rühmkorf (2015), Young (2013) and MacDonald (2011) discuss the collective dimensions to corporate harms and responsibilities and express their thoughts about how these dimensions might require new systems of liability, obligation and accountability (e.g. shared or network forms of liability). Lawrence et al. (2020) and Sjåfjell (2018) look at the options for ‘commoning’ the company and/or meaningfully changing the bounds of decision-making, ownership, benefit distribution, and participation.
